# Books and Magazines

**Published:** 1900-10

**Authors:** 


### Books and Magazines.

PANAMA AND THE SIERRAS.—A Doctor's Wander Days. By G. Frank Lydston, M. D. Price, $2.50, prepaid. The Riverton Press, 132 Market Street, Chicago.

This volume of about 250 pages, now in preparation, will comprise notes and sketches of several tours of Panama, the west coast of Central America and Mexico, and the most romantic regions of the Sierras. The book will be profusely and artistically illustrated from the author's original photographs, and will contain some unique and beautiful features in this respect. A special feature of the book will be its numerous character sketches of California pioneers, and observations of the Panama Canal. The publishers wish to announce a special author's edition, printed on fine deckle-edge paper and elegantly bound in cloth and gold of special design. The number of copies of this edition will be limited to the preliminary subscriptions, and will be signed and numbered.

Diabetis Mellitus—Its Detection and Successful Treatment.—The Journal has received a broachure under the above title, issued by the Charles Roome Parmele Co., 36 Platt Street, New York, the manufacturers and proprietors of Arsenauro and Murcano. (See advertisement on inside page.) It is a neatly printed pamphlet, containing very able and well written articles on the subject and illustrated with several colored plates showing the test for sugar in urine by the Fehling solution, and other cuts, Stern's Urino-Glucosometer and Einhorn's Saccharometer, Crystals of Phenyl Glucosazone as shown by Phenylhydrazin Test, etc. In the treatment of diabetis in a large number of cases reported in the papers referred to, and which are reprints from the *New York Medical Journal* (papers by Heinrich Stein, Ph. D., M. D., and Geo. D. Barney, M. D.), and from the *International Medical Magazine* (paper by Alexander W. Beck, M. D.), and several others, the two preparations above named were used, either exclusively or in connection with other remedies, and the results were remarkable. The Charles Roome Parmele Co. will mail a copy of this valuable pamphlet free to any one on receipt of a postal card, mentioning the "Red Back."

